# Insomnia and anxiety: exploring their hidden effect on natural killer cells among young female adults

**DOI:** 10.3389/fimmu.2025.1698155

**Published:** 2025-12-10

**Authors:** Renad M. Alhamawi, Fatmah A. Halawani, Sima F. Hakeem, Hadeel A. Alslimi, Ebraheim M. Alhamawi, Ahmed M. Aljohani, Ibrahim N. Mohammed, Heba M. Zahid, Yahya A. Almutawif

**Affiliations:** 1 Department of Clinical Laboratory Sciences, College of Applied Medical Sciences, Taibah University, Madinah, Saudi Arabia; 2 Health and Life Research Center, Taibah University, Madinah, Saudi Arabia; 3 Nujood Medical Center, Ministry of Health, Madinah, Saudi Arabia

**Keywords:** GAD, insomnia, young adults, NK cells, immune cells

## Abstract

**Introduction:**

Generalized anxiety disorder (GAD) is one of the most prevalent mental conditions globally, and it is frequently associated with sleep disturbances such as insomnia. Recently, these mental health conditions have been increasing in prevalence among younger generations, particularly among young women. Therefore, this study aimed to explore the relationship between anxiety, insomnia, and immune function, with a specific focus on natural killer (NK) cells.

**Methods:**

A cross-sectional study was conducted recruiting young female students under 25 years of age. Self-reported GAD-7 and insomnia symptoms were assessed using validated, previously published questionnaires, while immune cell profiles were measured by complete blood count (CBC) and flow cytometry.

**Results:**

The results revealed that 75% of participants experienced GAD-7 symptoms at varying severity levels, and over 50% reported insomnia. Interestingly, students with symptoms of GAD-7 had a lower percentage and number of circulatory NK cells and their subpopulation—CD16^+^CD56^dim^ and CD16^+^CD56^high^—compared to normal students. Moreover, among students who were suffering from insomnia, higher GAD-7 scores were negatively associated with the proportion of total peripheral NK cells.

**Discussion:**

These findings suggest that anxiety and sleep disturbances may compromise immune function and contribute to immune dysregulation. Raising awareness of these physiological effects may help in the prevention of inflammation-related diseases and cancers in young female populations.

## Introduction

Generalized anxiety disorder (GAD) is one of the most common mental conditions affecting many adults globally. The prevalence of GAD has increased dramatically over the last few decades, with women being more likely to be affected than men (1, 2). Young generations are found to develop high severity scores of GAD compared to the older population (3). GAD is characterized by persistent and excessive worries that interfere with daily life (4). GAD can associate with physiological symptoms including chest pain, dizziness, and shortness of breath (5). Recent studies have shown that anxiety-related disorders increase the risk of development of cardiovascular disease morbidity, such as coronary heart disease, heart failure, and stroke (6, 7). Indeed, this connection between mental health and physical resilience challenges suggests that anxiety is not just a mental issue but also a systemic one (8).

Sleeping difficulties such as insomnia are associated with anxiety among young adults aged under 25 years (9, 10). Insomnia is a condition characterized by difficulty falling asleep, staying asleep, or insufficient amount of sleep (11). It affects millions of people worldwide, especially women, causing multiple health issues (11). For example, insomnia is highly associated with heart diseases, cancer, and neurologic diseases (12–14). Therefore, understanding how GAD and insomnia disrupt physiological systems, particularly immune function, is essential, as this may help explain why individuals with anxiety and sleep disturbances exhibit increased susceptibility to infections and a heightened risk of developing chronic conditions, autoimmune disorders, and cancers.

The immune system is the pivotal system that protects the human body against invading pathogens and foreign bodies. It comprises two arms: innate and adaptive immunity. Innate immunity is a non-specific immunity constituting the first line of defense that comprises natural killer (NK) cells and phagocytic cells, such as neutrophils and monocytes (15, 16). Also, soluble mediators such as cytokines and the complement system play a crucial role in the innate part of the immune system (15). In contrast, adaptive immunity is a specific immunity that is divided into cellular and humoral immunity, composed of T lymphocytes and B lymphocytes, respectively (15, 16).

NK cells, also known as innate effector lymphocytes, are a group of large granular lymphocytes that make up 5%–15% of peripheral blood lymphocytes (17). They are crucial for immune surveillance as they can identify and destroy aberrant host cells without prior sensitization (17). NK cells are a heterogeneous population classified into CD16^+^CD56^dim^ cells, which constitute the majority of peripheral NK cells and exhibit cytotoxicity, and CD16^+^CD56^high^ cells that are less frequent but involved in cytokine production and immunoregulation (18, 19). Indeed, NK cells are well-defined immune effector cells that have a potent anti-viral and anti-tumor effects (20). Therefore, a deficiency in such cells might lead to immune system dysfunction, causing human diseases.

The incidence of anxiety and sleeping disorders has also risen over the past decade, particularly among the young population, especially young women (21–24). Given the limited data exploring the potential effects of anxiety and insomnia on the immune system, further research is warranted to assess the association between anxiety and insomnia, and the immune cells, including lymphocytes, monocytes, neutrophils, eosinophils, and basophils. Because of the potential role of NK cells in tumor and viral immunity, we focused on this study to explore the effect of these psychological conditions on such cells among the young female population.

## Materials and methods

### Study design and population

A total of 60 participants were recruited for this cross-sectional study at Taibah University in Madinah, Saudi Arabia, in February 2025. This study included young healthy female students aged between 17 and 23 years old. Participants with a medical history of chronic diseases such as diabetes, hypertension, and asthma were excluded from the study. Informed consent was obtained from all participants included in this study prior to data and sample collection. Research ethical approval was obtained from the research ethics committee at the University of Taibah in December 2024 (Project number 2025/209/103 MLT).

### Data collection

Data were collected during direct encounters between researchers and participants via a specially designed three-part questionnaire to ensure a high level of accuracy. The first part of the questionnaire that was developed for this study is detailed in the **Supplementary Materials**. This part collected the sociodemographic data, including the participant’s age, marital status, smoking behavior, physical activity, and caffeine consumption. It also included questions on the specialty of students, year of study, and grade point averages (GPAs).

The second questionnaire part assessed the level of anxiety symptoms using the validated Generalized Anxiety Disorder 7-item (GAD-7) questionnaire (25). The questionnaire included seven items that evaluated how frequently students experienced anxiety-related symptoms over the past 2 weeks. Each item was rated on a four-point scale: 0 (not at all), 1 (several days), 2 (more than half the days), and 3 (nearly every day). After collecting responses, individual scores for each item were added to calculate a total score for each student, with possible values ranging from 0 to 21. According to established cutoff points, scores were classified into four levels of anxiety severity: 0–4 (minimal anxiety), 5–9 (mild anxiety), 10–14 (moderate anxiety), and 15–21 (severe anxiety). Each participant self-reported anxiety symptoms using this questionnaire.

The third part focused on evaluating sleep difficulties. Participants completed a validated questionnaire consisting of eight items, known as the Sleep Condition Indicator (SCI) to assess insomnia and their sleeping pattern (26). Each item was rated on a 5-point scale from 0 to 4, with higher scores indicating better sleep quality. The total score ranged from 0 to 32. A lower total score reflected more severe sleep disturbance, while higher scores indicated healthier sleep patterns. A score below the cutoff (SCI ≤ 16) indicates a probable insomnia disorder. Participants who scored >16 were defined as normal, while those who scored ≤16 were defined as experiencing insomnia. Each participant self-reported insomnia symptoms using this questionnaire.

Students who did not self-report symptoms of GAD using the validated GAD-7 questionnaire and/or did not self-report symptoms of insomnia using the SCI questionnaire are referred to as “normal” in this study.

### Flow cytometry analysis

EDTA blood samples were collected from each participant. First, peripheral blood mononuclear cells (PBMCs) and polynuclear cells were isolated using RBC lysis buffer (Qiagen, USA). In brief, 1 mL was added to 500 μL of whole blood for 5–10 min at room temperature (RT), followed by washing with 1 mL of phosphate-buffered saline (PBS) and centrifuging for 5 min at 1,500 rpm. To exclude dead cells, the pellets were stained with Fixable Near-IR Dead Cell Stain (live/dead staining; Thermo Fisher Scientific Technology, USA) for 10 min at RT in a dark place, followed by washing using 1 mL of PBS and centrifuging for 5 min at 1,500 rpm. This stain was used to determine the viability of isolated leukocytes.

Then, 10 μL of the Triple-Color Reagent Anti-Human CD16/FITC Anti-Human CD56/RPE Anti-Human CD3/APC (Dako, Denmark) was added to each sample (resuspended in 50 μL) for 30 min at 4 °C. Then, cells were washed using 1 mL of PBS and centrifuged for 5 min at 1,500 rpm. Cells were re-suspended in 700 μL of PBS to run on the Attune Flow Cytometer (Thermo Fisher Scientific Technology, USA). Flow cytometry data were analyzed with FlowJo software (LLC, USA) version 10.

The percentage of NK cells and their subpopulation was determined using flow cytometry. Cells were pre-gated on leukocytes, singlets, and live cells before assessing the surface markers of NK cells. The number of NK cells and their subpopulation were determined from the total number of leukocytes by multiplying by the percentage of CD3^−^ cells and then multiplying the yielded number by the percentage of each subset of NK cells.

### Complete blood count analysis

EDTA blood samples were sent to Nujood Medical Center (Madinah, KSA) for complete blood count (CBC) assessment using the DxH 560 AL Hematology Analyzer (Beckman, USA). The report of each sample contained red blood cell (RBC) count, while blood cell (WBC) count, platelet count, hemoglobin (Hb) concentration, and the relative and absolute number of the following immune cells: lymphocytes, monocytes, neutrophils, eosinophils, and basophils.

### Statistical analysis

All statistical analyses and graphical representations were analyzed and created using GraphPad Prism version 10. Normality of distribution of all continuous variables was assessed using the Shapiro–Wilk test. Mann–Whitney test was performed to compare the mean across two different groups, while the Kruskal–Wallis test was used to compare the mean across more than two different groups. Spearman correlation was used to evaluate the strength of association between two continuous variables. Simple linear regression analysis was conducted to investigate the association between the total scoring of GAD-7 (independent variable) and frequencies and counts of circulating NK cell subsets (dependent variable). A 95% confidence level was applied to determine the significance of the data set.

## Results

### Subjects’ characterizations

**Figure 1:**
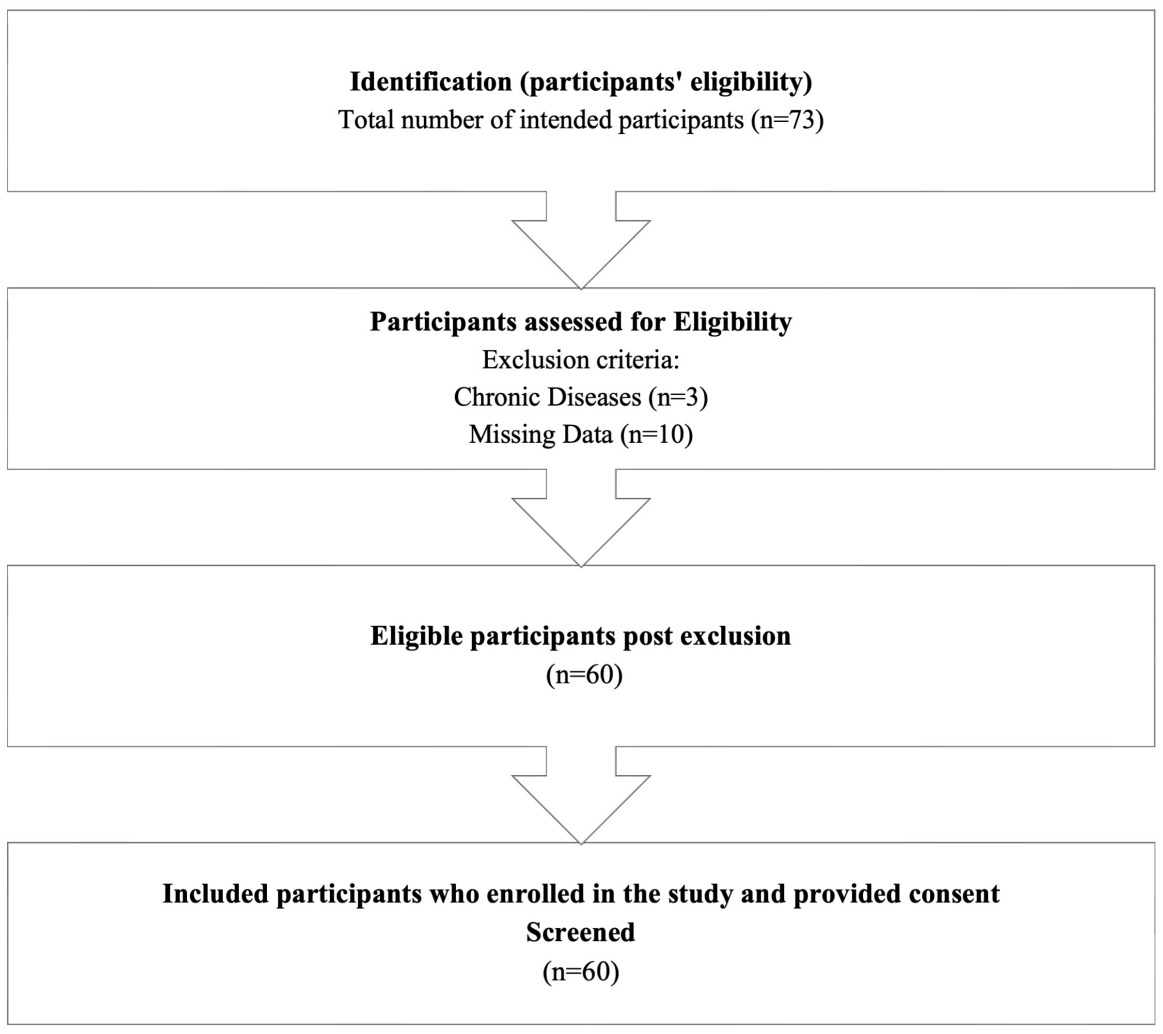
PRISMA flowchart for participants selection.

A total of 60 participants were included in this study based on inclusion and exclusion criteria illustrated in **Figure 1**. Among a total of 60 participants, approximately 42% were aged under 20, while 58% of students were aged above 20. The majority of participants were single (96.7%, *n* = 58). Approximately half of the students were not physically active, while 21.70% exercised twice a week. A considerable number of students consumed caffeine per day, 50% had one cup of coffee a day, while 28.3% had two to three cups of coffee per day, and 18.3% did not consume any caffeine. Approximately half of the participants specialized in Clinical Laboratory Sciences, 21.7% specialized in Pharmacology, and the remaining participants were from various other specialties such as Medicine, Chemistry, Cybersecurity, Data Science and Analytics, Law, and Management.

Moreover, 35% of participants were first-year students, 16.7% were second-year students, 25% were third-year students, and 23.3% were fourth-year students. The distribution of students’ GPAs is summarized as follows: 25% (*n* = 14) of the students had a GPA between 5 and 4.75, while 26.7% (*n* = 16) had a GPA ranging from 4.75 to 4.5. Additionally, 30% (*n* = 18) of the students reported a GPA between 4.5 and 4. A smaller proportion, 16.7%, had a GPA in the range of 4 to 3.5, and only 1.5% of students had a GPA between 3.5 and 3.0. Based on a questionnaire measuring the sleeping quality among students, more than half of the participants reported experiencing insomnia (53.30%, *n* = 32). Moreover, 75% of students were experiencing (GAD-7) based on their score. The severity of GAD-7 symptoms varied among students. Specifically, 45% of students had mild symptoms, while 16.7% and 13.3% exhibited moderate and severe symptoms, respectively. The characteristics of the participants are detailed in **Table 1**.

**Table 1:** Sample characteristics (*n* = 60).

	*n*	%
Age		
<20 years	25	41.70
≥20 years	35	58.30
Marital status		
Single	58	96.70
Married	1	1.70
Divorced	1	1.70
Smoking		
Yes	3	5.00
No	57	95.00
Physical activity per week		
Once	9	15.00
2 times	13	21.70
3–4 times	6	10.00
5–6 times	0	0.00
Daily	1	1.70
None	31	51.70
Caffeine consumption		
1 cup	30	50.00
2–3 cups	17	28.30
4–5 cups	1	1.70
>5 cups	1	1.70
None	11	18.30
Specialty		
Clinical laboratory sciences	31	51.70
Pharmacology	13	21.70
Medicine	1	1.70
Chemistry	4	6.70
Cybersecurity	1	1.70
Data science and analytics	4	6.70
Law	4	6.70
Management	2	3.30
Studying year		
1st year	21	35.00
2nd year	10	16.70
3rd year	15	25.00
4th year	14	23.30
GPA		
5–4.75	15	25.00
4.75–4.5	16	26.70
4.5–4	18	30.00
4–3.5	10	16.70
3.5–3	1	1.70
Sleep condition indicator (SCI)		
Normal (>16)	28	46.70
Experiencing insomnia (≤16)	32	53.30
GAD-7 scores		
Normal	15	25.00
Mild	27	45.00
Moderate	10	16.70
Severe	8	13.30

### Alteration of the CBC parameters and the proportion of NK cell subpopulations based on GAD-7 and insomnia stratification

**Figure 2:**
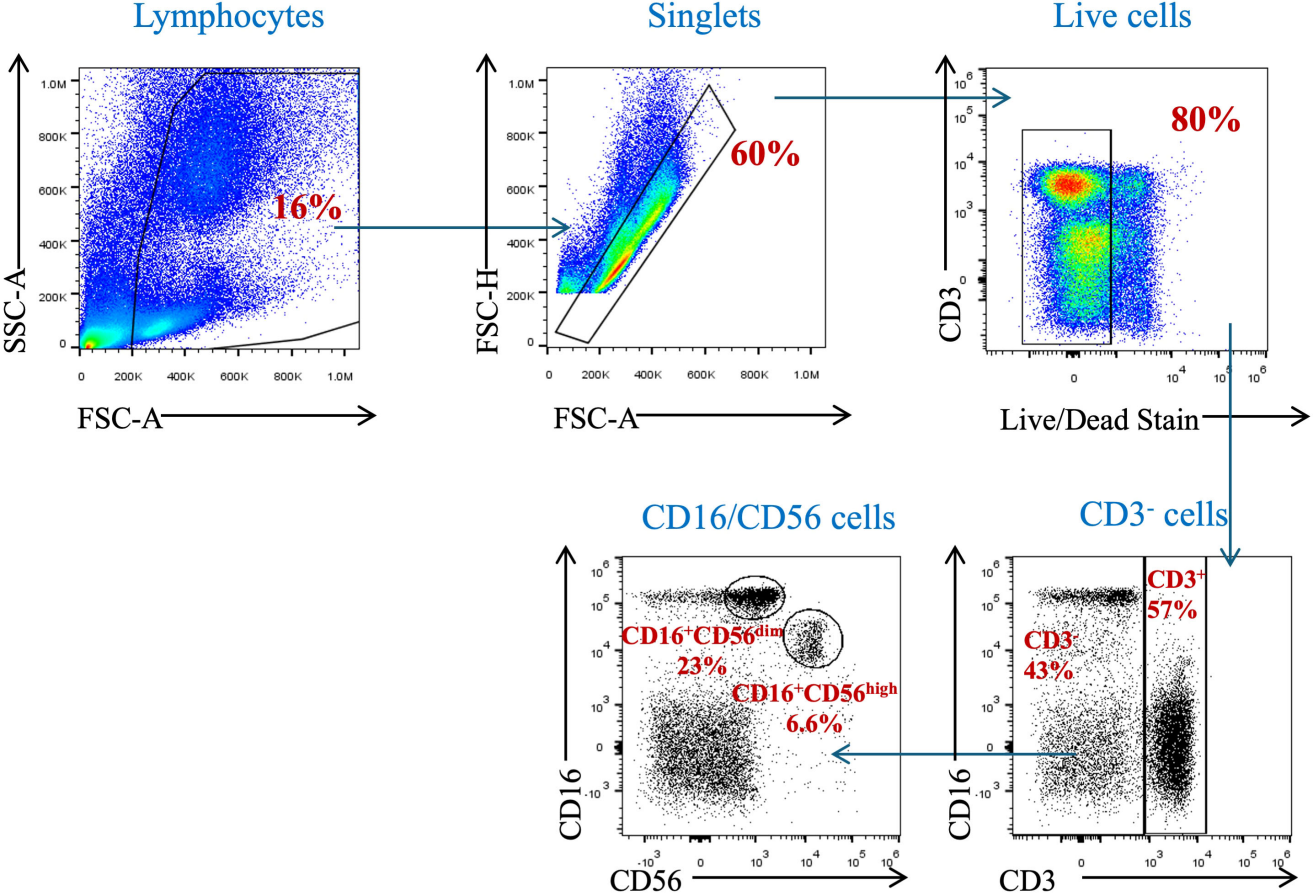
Gating strategy for the identification of natural killer cells and their subpopulations in peripheral blood. PBMCs isolated from whole blood were stained with anti-CD3, anti-CD16, and anti-CD56 mAbs. Cells were pre-gated on live, single, and CD3^−^ cells before identifying the NK cell population (CD3^−^CD16^+^CD56^+^ cells).

To assess the proportion of circulating NK cells, a gating strategy was set up to identify such cells in the participants’ peripheral blood samples. Using the surface markers, CD3, CD16, and CD56, we identified NK cells among PBMCs and polynuclear cells. Leukocytes were identified due to their forward and sideward scatter profile. Dead cells were excluded by staining them with live/dead stain. NK cells were identified as being CD3-negative and CD16- and CD56-positive cells (**Figure 2**).

The proportion and absolute number of peripheral NK cells and CBC parameters were evaluated according to GAD-7 and insomnia. Students who reported experiencing insomnia had a higher hemoglobin concentration compared to normal students; however, the value remained within normal range (**Table 2**). Students who reported experiencing insomnia had a significant lower proportion of CD16^+^CD56^high^ NK compared to normal students (*p* = 0.022, **Table 2**). However, all other parameters showed a similar association with insomnia detailed in **Table 2**.

**Table 2:** Association between sleeping quality and CBC parameters and NK cells and their population.

		Normal (*n* = 28)	Insomnia (*n* = 32)	*P*-value
WBCs ×10^3^		6.76 ± 2.2	6.64 ± 2	0.997
RBCs ×10^6^		4.6 ± 0.3	4.8 ± 0.36	0.089
Platelets ×10^3^		322 ± 82.73	310.1 ± 69.18	0.656
Hemoglobin (g/dL)		12.6 ± 1.2	13.3 ± 1.2	0.039*
Lymphocytes	**Percentage**	36.4 ± 11.6	35.8 ± 6.8	0.752
	**Count (×10^3^)**	2.3 ± 0.7	2.4 ± 0.7	0.505
Monocytes	**Percentage**	7.5 ± 2.3	7.4 ± 1.9	0.904
	**Count (×10^3^)**	0.48 ± 0.15	0.5 ± 0.13	0.811
Neutrophils	**Percentage**	53.7 ± 13.1	54.3 ± 8.6	0.974
	**Count (×10^3^)**	3.8 ± 1.9	3.7 ± 1.2	0.738
Eosinophils	**Percentage**	2 ± 1.5	2.04 ± 1.4	0.783
	**Count (×10^3^)**	0.12 ± 0.09	0.13 ± 0.9	0.683
Basophils	**Percentage**	0.4 ± 0.2	0.38 ± 0.09	0.968
	**Count (×10^3^)**	0.03 ± 0.01	0.26 ± 0.01	0.819
Total NK cells of CD3^−^ cells	**Percentage**	27.5 ± 16.2	22.03 ± 19.02	0.163
	**Count**	824.6 ± 645.4	664.1 ± 722.3	0.212
CD16^+^CD56^dim^ cells of CD3^−^ cells	**Percentage**	20.2 ± 13.6	17.5 ± 16.4	0.258
	**Count**	589.4 ± 480	521.5 ± 577.9	0.285
CD16^+^CD56^high^ of CD3^−^ cells	**Percentage**	7.4 ± 5.6	4.5 ± 3.9	0.022*
	**Count**	235.2 ± 250.5	142.5 ± 170.3	0.067

*<0.05, **<0.01.

**Figure 3:**
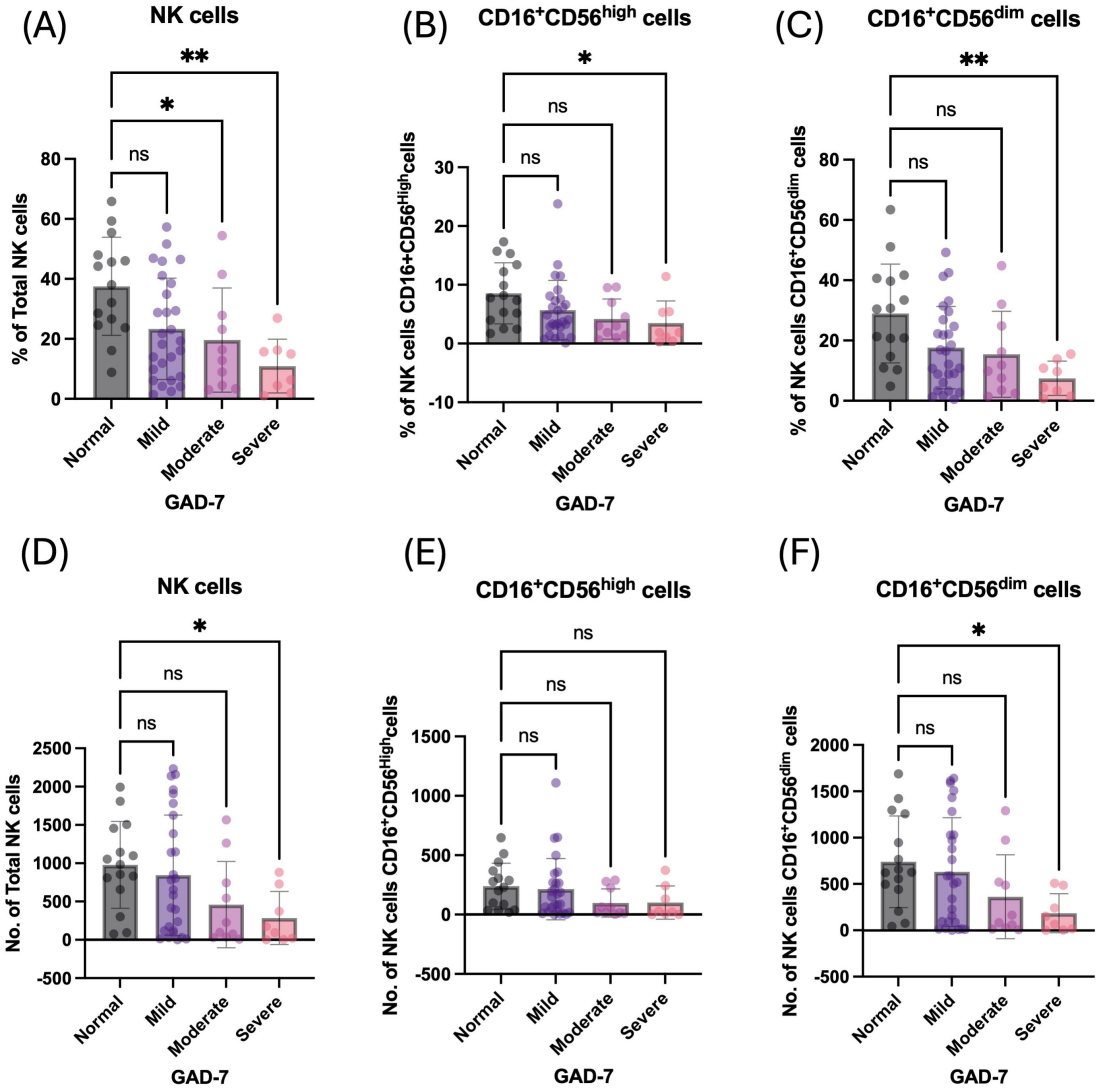
Anxiety has a negative influence on the percentage of peripheral NK cells and their subpopulation. **(A)** Percentage of total NK cells based on GAD-7 categories; mild, moderate, and severe compared to normal. **(B)** Percentage of CD16^+^CD56^dim^ NK cells based on GAD-7 categories; mild, moderate, and severe compared to normal. **(C)** Percentage of CD16^+^CD56^high^ NK cells based on GAD-7 categories; mild, moderate, and severe compared to normal. **(D)** Absolute cell number of total NK cells based on GAD-7 categories; mild, moderate, and severe compared to normal. **(E)** Absolute cell number of CD16^+^CD56^dim^ NK cells based on GAD-7 categories; mild, moderate, and severe compared to normal. **(F)** Absolute cell number of CD16^+^CD56^high^ NK cells based on GAD-7 categories; mild, moderate, and severe compared to normal. ns, non-significant, *<0.05, **<0.01.

On the other hand, students with symptoms of GAD-7 had a significantly lower percentage and number of circulatory NK cells and their subpopulations: CD16^+^CD56^dim^ and CD16^+^CD56^high^ compared to normal students (**Table 3**). The percentage of total NK cells was negatively correlated with increasing symptoms of GAD-7, showing the lowest percentage of such cells among students with severe symptoms (*p* = 0.0018; **Figure 3A**). It has also been observed in the absolute number of total NK cells (**Figure 3D**). The percentage of NK cell subpopulations, CD16^+^CD56^dim^ and CD16^+^CD56^high^ cells, were lower in participants with severe symptoms of GAD-7 compared to normal participants (**Figures 3B, C**). However, the absolute number of CD16^+^CD56^dim^ cells only were lower in students with GAD-7 symptoms compared to normal students (**Figures 3E, F**). Taken together, the levels of anxiety and sleeping disturbance may have a negative correlation with the proportion of NK cells among young students.

**Table 3:** Association between GAD-7 and CBC parameters and NK cells and their population.

		Normal (*n* = 15)	Mild (*n* = 27)	Moderate (*n* = 10)	Severe (*n* = 8)	*p*-value
WBCs ×10^3^		6.7 ± 2.8	6.9 ± 1.8	6.8 ± 1.9	5.8 ± 1.6	0.456
RBCs ×10^6^		4.6 ± 0.36	4.6 ± 0.3	4.8 ± 0.31	4.9 ± 0.45	0.285
Platelets ×10^3^		306.6 ± 75.1	316 ± 87.8	310.2 ± 63.1	388.2 ± 4.3	0.628
Hemoglobin (g/dL)		12.7 ± 1	13.1 ± 1.3	12.6 ± 1.3	13.5 ± 1.3	0.410
Lymphocytes	**Percentage**	35.3 ± 12.2	36.5 ± 9.2	34.3 ± 6	38.4 ± 7.4	0.708
	**Count (×10^3^)**	2.4 ± 0.75	2.4 ± 0.64	2.3 ± 0.9	2.1 ± 0.3	0.614
Monocytes	**Percentage**	7.5 ± 2.3	7.1 ± 2	7.6 ± 1.1	8.4 ± 2.8	0.709
	**Count (×10^3^)**	0.5 ± 0.13	0.5 ± 0.16	0.5 ± 0.13	0.46 ± 0.12	0.852
Neutrophils	**Percentage**	54.6 ± 14.6	54.2 ± 10	55.8 ± 6.1	50 ± 11	0.715
	**Count (×10^3^)**	4.1 ± 2.1	3.9 ± 1.4	3.7 ± 1.1	3 ± 1.3	0.501
Eosinophils	**Percentage**	2.3 ± 1.9	1.7 ± 1	1.8 ± 1	3 ± 1.9	0.337
	**Count (×10^3^)**	0.14 ± 0.1	0.12 ± 0.07	0.11 ± 0.05	0.17 ± 0.1	0.666
Basophils	**Percentage**	0.31 ± 0.09	0.43 ± 0.2	0.4 ± 0.1	0.4 ± 0.1	0.07
	**Count (×10^3^)**	0.023 ± 0.01	0.03 ± 0.01	0.03 ± 0.01	0.02 ± 0.004	0.212
Total NK cells of CD3^−^ cells	**Percentage**	37.5 ± 16.4	23.3 ± 16.9	19.6 ± 17.3	10.9 ± 9	0.004*
	**Count**	978 ± 567	844.3 ± 784.9	459.5 ± 563.6	285 ± 346	0.027*
CD16^+^CD56^dim^ cells of CD3^−^ cells	**Percentage**	29 ± 16.4	17.6 ± 13.7	15.4 ± 14.3	7.5 ± 5.7	0.009**
	**Count**	740 ± 494	629.8 ± 586.1	362.2 ± 452.6	184 ± 210	0.026*
CD16^+^CD56^high^ of CD3^−^ cells	**Percentage**	8.5 ± 5.2	5.7 ± 5.1	4.2 ± 3.4	3.5 ± 3.8	0.034*
	**Count**	238 ± 193.7	214.5 ± 258.2	97.3 ± 117.7	100.5 ± 139.5	0.071

*<0.05, **<0.01.

### Association of NK cell subsets and GAD-7 scoring and sleeping quality scoring based on insomnia scores

It was important to assess whether GAD-7 scoring could impact NK cells and their subsets among students based on sleeping status (normal and who reported insomnia). The Spearman correlation analysis was conducted to assess the correlation between sleeping quality scoring and GAD-7 scoring and the proportion and count of circulating NK cells and their subsets based on insomnia score (SCI) stratification. The analysis indicated a significant negative moderate correlation between the proportion and count of both total NK cells and the CD16^+^CD56^dim^ subset and GAD-7 scoring only among the participants who reported experiencing insomnia (*r*s = −0.457, *r*s = −0.439, *r*s = −0.474, and *r*s = −0.446, respectively; **Table 4**).

**Table 4:** Correlation between frequencies of peripheral natural killer cells and their subpopulations in peripheral blood and age, the total score of sleeping quality, and the total score of GAD-7.

		Total score of GAD-7	Total score of sleep condition indicator
Normal (SCI > 16) students (*n* = 28)			
Total NK cells of CD3^−^ cells	**Percentage**	−0.261	0.160
	**Count**	−0.195	0.225
CD16^+^CD56^dim^ cells of CD3^−^ cells	**Percentage**	−0.214	0.133
	**Count**	−0.143	0.204
CD16^+^CD56^high^ of CD3^−^ cells	**Percentage**	−0.257	0.205
	**Count**	−0.204	0.283
Students experiencing insomnia (SCI ≤ 16) (*n* = 32)			
Total NK cells of CD3^−^ cells	**Percentage**	−0.457**	0.003
	**Count**	−0.439*	−0.039
CD16^+^CD56^dim^ cells of CD3^−^ cells	**Percentage**	−0.474**	0.040
	**Count**	−0.446*	−0.026
CD16^+^CD56^high^ of CD3^−^ cells	**Percentage**	−0.263	−0.052
	**Count**	−0.290	−0.112

*<0.05, **<0.01.

**Figure 4:**
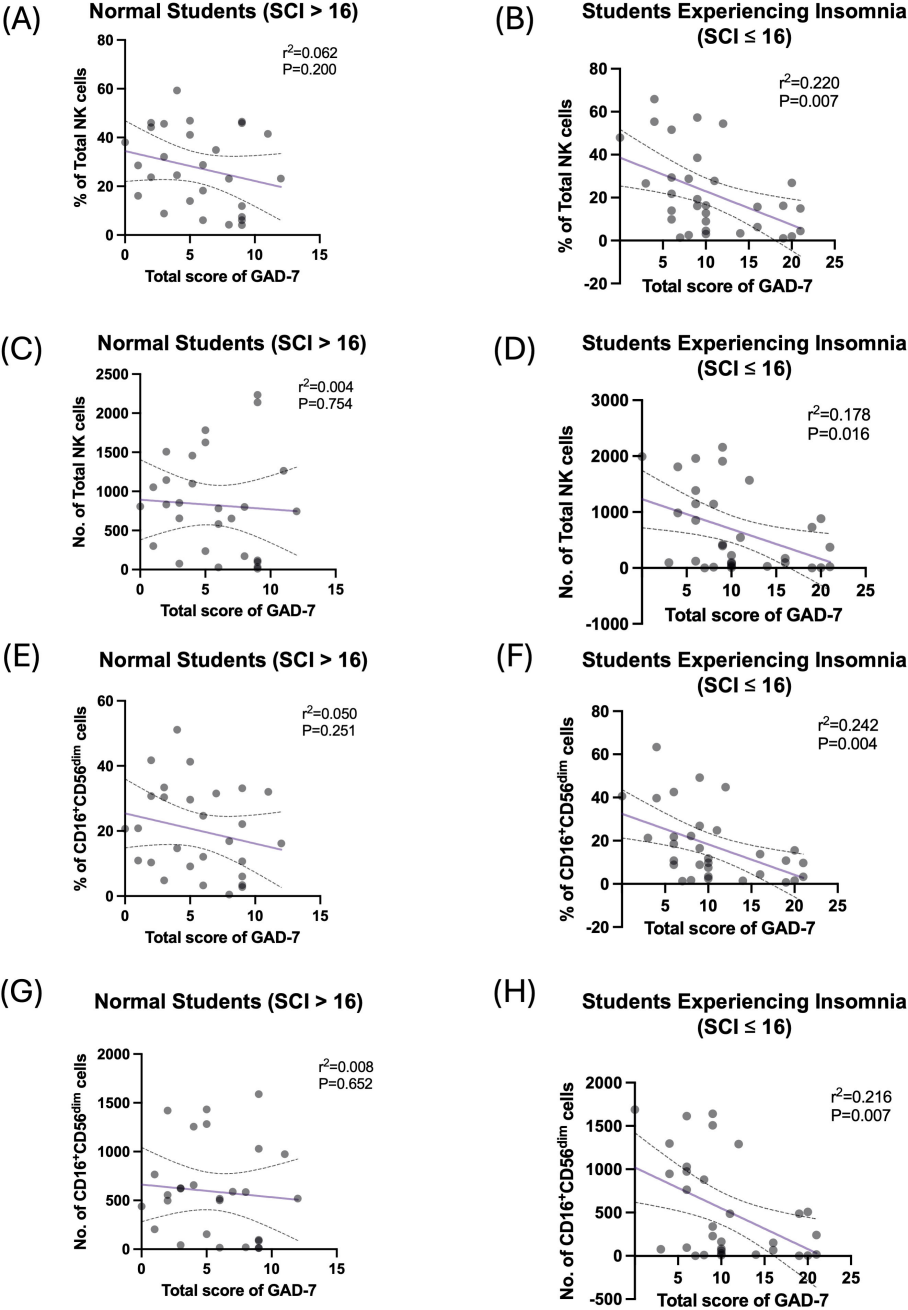
High GAD-7 scores are negatively correlated with circulating NK cells in the students who reported experiencing insomnia. **(A)** Simple linear regression between percentage of total NK cells and scoring of GAD-7 in normal (SCI > 16) participants. **(B)** Simple linear regression between percentage of total NK cells and scoring of GAD-7 in participants who reported experiencing insomnia (SCI ≤ 16). **(C)** Simple linear regression between number of total NK cells and scoring of GAD-7 in normal (SCI > 16) participants. **(D)** Simple linear regression between number of total NK cells and scoring of GAD-7 in participants who reported experiencing insomnia (SCI ≤ 16). **(E)** Simple linear regression between percentage of CD16^+^CD56^dim^ NK cells and scoring of GAD-7 in normal (SCI > 16) participants. **(F)** Simple linear regression between percentage of CD16^+^CD56^dim^ NK cells and scoring of GAD-7 in participants who reported experiencing insomnia (SCI ≤ 16). **(G)** Simple linear regression between number of CD16^+^CD56^dim^ NK cells and scoring of GAD-7 in normal (SCI > 16) participants. **(H)** Simple linear regression between number of CD16^+^CD56^dim^ NK cells and scoring of GAD-7 in participants who reported experiencing insomnia (SCI ≤ 16).

Multivariate regression analysis was performed to explore the strength of association between the total score of GAD-7 and NK cells and their subsets (**Table 5**). Among the students who reported experiencing insomnia, GAD-7 scoring predicted a smaller proportion of total NK cell frequency and count, wherein GAD-7 scoring explained 22% and 17.8% of the change in proportion and number of total NK cells, respectively (**Figures 4A–D**). Moreover, GAD-7 scoring inversely affected the proportion and number of peripheral CD16^+^CD56^dim^ NK cells found only among students suffering from insomnia (**Figures 4E–H**). The regression coefficient for the percentage was β = –1.412 (SE = 0.457, 95% CI: –2.345 to –0.479, *R*^2^ = 0.242, *p* = 0.004). Similarly, the absolute count of CD16^+^CD56^dim^ cells showed a significant inverse relationship, with β = –47.080 (SE = 16.388, 95% CI: –80.549 to –13.611, *R*^2^ = 0.216, *p* = 0.007).

**Table 5:** Multivariate regression analysis for the association between the total score of GAD-7 and CBC parameters and NK cells and their population.

		*B*	SE	95% Confidence interval	*R* ^2^	*P*-value
Normal (SCI > 16) students (*n* = 28)						
Total NK cells of CD3^−^ cells	**Percentage**	−1.227	0.933	−3.145 to 0.692	0.062	0.200
	**Count**	−12.180	38.386	−91.082 to 66.723	0.004	0.754
CD16^+^CD56^dim^ cells of CD3^−^ cells	**Percentage**	−0.929	0.792	−2.556 to 0.698	0.050	0.251
	**Count**	−12.981	28.475	−71.511 to 45.549	0.008	0.652
CD16^+^CD56^high^ of CD3^−^ cells	**Percentage**	−0.298	0.326	−0.968 to 0.373	0.031	0.370
	**Count**	0.801	14.924	−29.875 to 31.478	0.000	0.958
Students experiencing insomnia (SCI ≤ 16) (*n* = 32)						
Total NK cells of CD3^−^ cells	**Percentage**	−1.565	0.538	−2.664 to −0.467	0.220	0.007**
	**Count**	−53.470	20.970	−96.296 to −10.643	0.178	0.016*
CD16^+^CD56^dim^ cells of CD3^−^ cells	**Percentage**	−1.412	0.457	−2.345 to −0.479	0.242	0.004**
	**Count**	−47.080	16.388	−80.549 to −13.611	0.216	0.007**
CD16^+^CD56^high^ of CD3^−^ cells	**Percentage**	−0.153	0.123	−0.403 to 0.098	0.49	0.222
	**Count**	−6.390	5.327	−17.269 to 4.489	0.046	0.240

*<0.05, **<0.01.

## Discussion

This study was set up to explore the harmful effects of anxiety and sleep disturbance on the immune system, particularly NK cells, which might lead to the development of inflammatory diseases and cancers. In this cross-sectional study, we found that approximately 75% of female students reported GAD at different levels of severity, and 50% reported sleeping disturbance suggestive of insomnia. This observation is consistent with a previously published study in the region showing that among female students, 68% were diagnosed with GAD (24). Indeed, a national screening study conducted in Saudi Arabia found that the prevalence of GAD has increased significantly, especially among women (23). Moreover, another study assessing the prevalence of insomnia in the Saudi population using the same tool employed in this study found that approximately 38% of the population suffered from insomnia, particularly among women, students, and unemployed individuals (22). A recent systematic review and meta-analysis evaluating the prevalence of insomnia among university students in Saudi Arabia reported a noticeable rise in insomnia, especially among medical students and female students (27).

The increasing burden of anxiety and sleep-related disorders raises important questions regarding their potential consequences for overall human health and disease risk. Thus, this study was focused on investigating the influence of GAD-7 and insomnia on the proportion and number of peripheral immune cells, particularly NK cells. Interestingly, a significant reduction of NK cells was observed among participants experiencing severe GAD-7 symptoms. The negative association between the frequency and number of circulating NK cells and the total scores of GAD-7 was observed among students who reported experiencing insomnia. These findings are consistent with the literature, as it has been shown that patients with chronic tinnitus had a negative association between stress level and the frequency of CD16^+^CD56^dim^ NK cells (28). Anxiety can strongly influence not only the number and proportion of NK cells but also the functionality of such cells (29). A reduction in the number and/or functional activity of NK cells is strongly associated with cancer pathogenesis and is frequently linked to poor prognosis (30–32). Therefore, alteration of NK cell number and activity due to anxiety-related disorders might increase the risk of cancer development.

Moreover, a reduction of NK cells was associated with other psychological disorders such as depression (33, 34). It has been shown that hospitalized patients with depression had a decreased activity of NK cells compared to healthy controls; however, this was not the case in patients with schizophrenia (33, 34). Additionally, the proportion of peripheral NK cells was significantly decreased among patients suffering from major depressive disorders (35). Collectively, mental disorders, including depression and anxiety, might influence the immune system, affecting both number and function of NK cells.

Indeed, anxiety-related disorders can affect the immune system in either a direct or an indirect manner. For example, it has been shown that the salivary cortisol significantly increased among anxious subjects and was positively associated with the total score of GAD (36). Moreover, a Mendelian randomization study has demonstrated the positive association between cortisol level and anxiety, which is associated with high blood pressure (37). To this end, anxiety-related disorders are strongly associated with elevated levels of the cortisol hormone, suggesting that anxiety may indirectly suppress the immune system through prolonged activation of the stress response.

Cortisol is a glucocorticoid hormone produced by the adrenal cortex, which is known as an immunosuppressive agent (38). It has been shown that long-term treatment with glucocorticoids was associated with a significant reduction and impaired function of CD4^+^ T cells, leading to a severe decline in CD4/CD8 ratio (39). High level of cortisol exposure is also associated with a decreased number of circulatory B lymphocytes among students (40). Moreover, a study showed that anxiety in cancer patients is associated with a high level of cortisol, leading to decreased NK cell activity and an increase in the level of pro-inflammatory cytokines that are linked to poor prognosis and impaired treatment efficiency (41). Collectively, stress and high cortisol levels are strongly associated with impaired function of the immune system.

Studying the interaction between emerging psychotically health problems such as GAD and insomnia, and the immune system is crucial to understand the development of occurrence of inflammatory diseases and cancer incidence. To our knowledge, this is the first study in the region to assess the impact of GAD and insomnia on the immune system among young adults. However, this study is limited by the inclusion of young women only, which results in limited generalizability of the findings. Further studies should be conducted across different age groups, such as children and older adults. A larger sample size should be considered, including a diverse population from different regions, including both male and female patients, to extend the overall view of the hidden effects of GAD and insomnia on the proportion and function of immune cells. Assessing different inflammatory markers, such as C-reactive protein (CRP) and cortisol hormone, would give depth to the study, in which they are correlated with total scores of GAD and insomnia and the proportion of peripheral immune subsets. Also, future studies can include an *in vitro* functional assay of different immune cells based on GAD and insomnia stratification.

## Conclusion

GAD is one of the most prevalent mental conditions globally, and it is frequently associated with sleep disturbances such as insomnia. These mental health conditions are developing among the younger generation, particularly the young female population. These disorders can disrupt the normal functioning of various human body systems, including the immune system, thereby contributing to the development of chronic and inflammatory diseases. Such impacts ultimately compromise overall health and quality of life. Accordingly, there is an urgent need to investigate how anxiety and sleep disturbance impact immune function. Understanding how these psychological stressors influence the distribution and activity of immune cells, especially peripheral NK cells, may provide valuable insights into the mechanisms underlying inflammation and tumorigenesis. These findings could support the development of novel strategies to raise awareness about the physiological consequences of anxiety and insomnia, ultimately helping in the prevention of immune-related disorders and cancers.

## Data Availability

The original contributions presented in the study are included in the article/**Supplementary Material**. Further inquiries can be directed to the corresponding author.
