# Supplementary material for: Insomnia and anxiety: exploring their hidden effect on natural killer cells among young female adults
Source: Front Immunol. 2025 Dec 10;16:1698155. doi: 10.3389/fimmu.2025.1698155 (PMC12728036; doi:10.3389/fimmu.2025.1698155)
Supplement: Supplementary file 1 [file Table1.docx]

Supplementary materials

Questionnaire in English:

Q1- How old are you?

Answer (………….)

Q2- What is your marital status?

1. Single
2. Married
3. Divorced

Q3- Do you smoke?

1. Yes
2. No

Q4-If the answer is yes, what type of smoking?

1-Cigarettes

2-E-cigarettes

3-Hookah

Q5-Are you following any specific diet?

1. Yes
2. No

Q6-If the answer is yes, what type of diet?

Answer (………….)

Q7-How often do you exercise per week?

1. Once a week
2. Twice a week
3. 3 - 4 times a week
4. 5-6 times a week
5. Everyday
6. Never

Q8-How many cups of coffee consume per day?

1. One cup
2. 2 - 3 cups
3. 4 - 5 cups
4. More than 5 cups
5. None

Q9-Are you suffering from any chronic conditions?

1-Yes (…………)

2-No

Q10- Do you use any medical medications? If yes specify?

1-Yes (…………)

2-No

Q11-Do you use any Non-Medical Medications? If yes specify?

1-Yes (…………)

2-No

Q12-What is your specialty?

Answer (………….)

Q13-Which academic year are you in?

1. 1st year
2. 2nd year
3. 3rd year
4. 4th year
5. 5th year
6. 6th year

Q14-What is your GPA?

Answer (………….)

Q15- What is your GPA?

1. 5 – 4.75
2. 4.75 – 4.50
3. 4.50 – 4
4. 4 – 3.50
5. 3.50 – 3
6. 3 – 2.50
7. 2.50 – 1
